# Multiple Thromboses in a Patient with Systemic Lupus Erythematosus after Splenectomy

**DOI:** 10.1155/2012/813629

**Published:** 2012-05-28

**Authors:** Deng-Ho Yang

**Affiliations:** ^1^Division of Rheumatology/Immunology/Allergy, Department of Internal Medicine, Armed-Forces Taichung General Hospital, Number 348, Section 2, Chung Shan Road, Taiping District, Taichung City 411, Taiwan; ^2^Institute of Medicine, Chung Shan Medical University, Taichung city, Taiwan

## Abstract

Antiphospholipid syndrome is a disorder presenting with arterial or venous thrombus and a history of fetal loss. Early diagnosis and adequate treatment is important to prevent multiple organ failures. Here, we described a woman with a two-year history of systemic lupus erythematosus with severe nephrotic syndrome, manifested multiple thrombi over the portal vein and the inferior vena cava, combined with acute renal infarction. The patient underwent splenectomy 10 months ago. Initially, she received anticoagulant treatment and low-dose glucocorticoid, but multiple organ failure progressed. After emergency plasma exchange followed by glucocorticoid pulse therapy, the patient recovered.

## 1. Introduction

Systemic lupus erythematosus (SLE) is an autoimmune disease with multiple organ involvement and is a common cause of secondary antiphospholipid syndrome (APS). APS is defined by arterial or venous thrombus, recurrent fetal loss, and thrombocytopenia with positive antibodies including lupus anticoagulant (LA), anticardiolipin antibodies (aCL), and antibodies to *β*2-glycoprotein-I (anti-*β*2GPI) [1, 2]. Thrombus-induced various organ infarctions such as deep vein thrombosis, stroke, pulmonary embolism, bowel, or heart ischemia, which are common in SLE patients with secondary APS. Here, we report a patient with SLE and secondary APS with coexisting renal infarction and a large thrombus over the portal vein and the inferior cava.

## 2. Case Report

**Figure 1:**
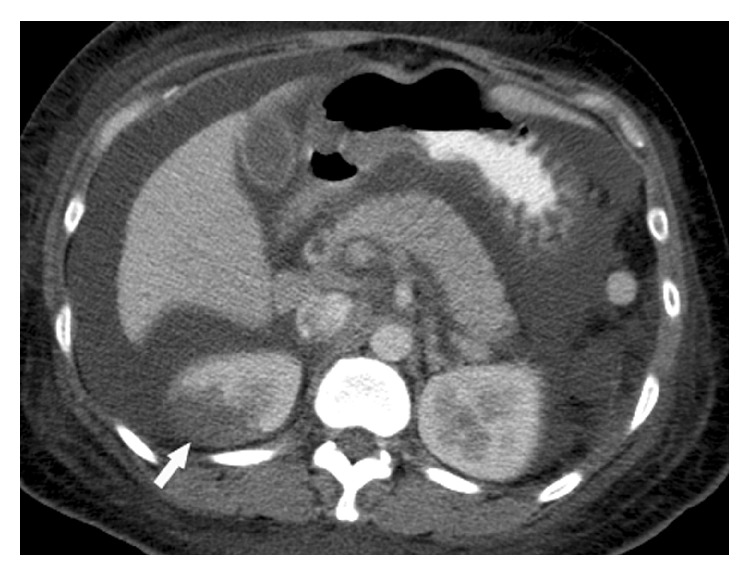
The axial contrast-enhanced CT image showing a wedge-shaped infarction (arrow) involving the right kidney.

**Figure 2:**
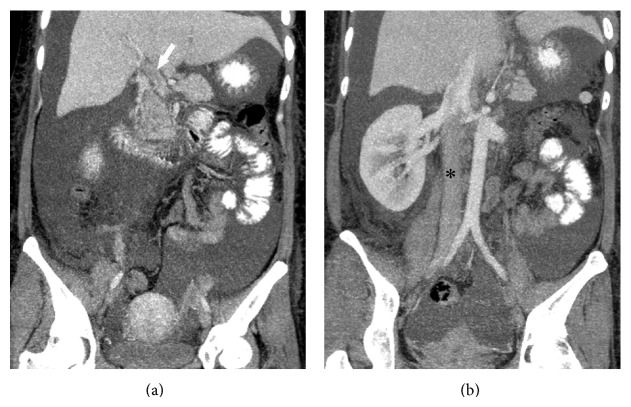
The reformatted coronal CT image showing (a) thrombosis of the main portal vein (arrow), and (b) a long segmental thrombus in the inferior vena cava (∗).

In January 2006, a 33-year-old woman was diagnosed with SLE, based on malar rash, positive ANA (1 : 640, mixed pattern), high titer of anti-dsDNA (140 IU/mL, normal <10), and autoimmune hemolytic anemia. Since then, she received immunosuppressive medications including prednisolone, azathiopurine, and hydroxychloroquine. In January 2007, splenectomy was performed on account of refractory hemolytic anemia and thrombocytopenia. In June 2007, severe nephritic syndrome with urine daily protein loss (DPL) 8 g developed. She received renal biopsy, and the biopsy revealed membranous glomerulonephritis. Monthly pulses of cyclophosphamide combined with pulse corticosteroids therapy was initiated thereafter; however, the response was poor. Persistent proteinuria (urine DPL: 5 to 10 g) was still found. In November 2007, she presented with intermittent abdominal pain in the emergency room. Physical examination revealed decreased bowel sound, positive shifting dullness, rebounding tenderness in the right lower quadrant, left costovertebral-angle tenderness, and peripheral bilateral leg edema. Laboratory data revealed the following results: WBC, 4,700/mm^3^ (normal 4,500–11,000); hemoglobin, 13.3 g/dL (normal 12–16); platelets, 179,000/mm^3^ (normal 150,000–400,000); BUN, 23 mg/dL (normal 7–20); creatinine, 0.4 mg/dL (normal 0.5–1); alanine aminotransferase, 28 U/L (normal <31); aspartate transaminase, 18 U/L (normal <31); albumin, 1.7 g/dL (normal 3.4–4.8); d-dimer, 3,516 ng/mL (normal <500); fibrinogen, 933 mg/dL (normal 200–400). The urine DPL was 10.3 g. Immunological studies were as follows: positive LA; aCL-IgG, 55 U/mL (normal <15); aCL-IgM, 19.6 U/mL (normal <15); anti-dsDNA, 394 IU/mL; C3, 48.8 mg/dL (normal 90–180); C4, 16.8 mg/dL (normal 10–40), and the serological test for syphilis was negative. Antithrombin III was 59% (normal 70–120). Multidetector-row computed tomography (MDCT) demonstrated a wedge-shaped infarction involving the right kidney (Figure 1), as well as segmental thrombus in the inferior vena cava (IVC) and main portal vein (Figure 2). Initially, the patient was managed with anticoagulants (low-molecular-weight heparin) and intravenous methylprednisolone 250 mg daily for three days. However, right lower quadrant abdominal pain with acute renal failure (serum creatinine: 2.5 mg/dL) progressed. Elevation of anti-dsDNA (412 IU/mL) and reduction of C3 and C4 (40 and 9 mg/dL) were also found from following laboratory data. Acute SLE flare with acute renal failure and multiple thrombus events was impressed. Due to poor response for anticoagulant and methylprednisolone therapy, plasma exchange was carried out 5 times by using fresh frozen plasma as the replacement fluid, followed by corticosteroids pulse therapy (1,000 mg intravenous methylprednisolone for 3 days). After therapy, she recovered and was discharged on the 14th hospital day with normal d-dimer and a targeted international normalized ratio (INR) of 2.5. The patient's renal functions were within normal range. Decreased serum levels of anti-dsDNA, and increased C3 and C4 was also found. The patient had no recurrent symptoms of thrombus or emboli in the following three months. Until now, she visited outpatient department regularly and received medication including prednisolone, hydroxychloroquine, and azathiopurine.

## 3. Discussion

APS is an autoimmune disorder defined as the presence of antiphospholipid antibodies (aPLs) with arterial or venous thrombosis, recurrent spontaneous abortions, and thrombocytopenia. aPLs include aCL, LA, and anti-*β*2GPI, but small number is seronegative APS [2]. Approximately, half of the primary APS cases and one third of the secondary APS cases are associated with SLE. Other conditions that are associated with secondary APS include lupus-like syndrome, primary Sjogren's syndrome, rheumatoid arthritis, systemic sclerosis, systemic vasculitis, and dermatomyositis [1]. Most cases of APS present peripheral or pulmonary thrombosis and neurological manifestations, whereas intra-abdominal involvement is uncommon. Hepatic involvement is the most common abdominal manifestation in APS [1, 3, 4]. Intestinal infarction due to thrombosis of mesenteric vessels or IVC is infrequently reported.

Catastrophic APS (CAPS) is a variant form of APS and predominantly a small vessel occlusive disease mainly affecting parenchymal organs resulting in multiple organ failure, and its prevalence is less than 1% [5]. CAPS has a high mortality rate in the absence of aggressive and emergency therapy. Our patient had coexisting acute renal infarction and thrombosis of IVC and portal vein. Multiple large vessel involvement is different from the classical manifestation of CAPS which consists of multiple thromboses of medium and small vessels. However, multiple organ involvement including that of the liver, kidney, and intestine was observed in this case. The patient had membranous type of lupus nephritis with severe proteinuria. High incidence of thromboembolic complications is observed in patients with nephrotic syndrome, and most cases have venous events including renal, pulmonary, and deep-vein thromboses [6, 7]; however, arterial thrombosis-associated nephrotic syndrome is uncommon. The pathogenesis of thrombotic abnormalities in the nephritic syndrome includes increased platelet hyperaggregability, hyperfibrinogenemia, and decreased antithrombin III [6, 7]. From the laboratory data, our patient had significantly reduction of antithrombin III levels and elevation of fibrinogen levels. Therefore, the presentation of severe nephrotic syndrome may be one of the risk factors to develop IVC thrombosis in this case.

Most patients who undergo splenectomy are at an increased risk of developing portal system thrombosis within 1 month after the operation, but a few cases have late presentation (13–46 months postoperatively) [8]. Portal system thrombosis may be asymptomatic. Our patient received splenectomy 10 months ago, and we considered that her portal vein thrombosis may be associated with the operation of splenectomy. In this case, multiple factors were found to trigger thromboembolic complications including APS, poor control of nephrotic syndrome, and splenectomy.

Current therapies for APS include heparin (low-molecular-weight and unfractionated heparin), warfarin, antiplatelet agent (aspirin and clopidogrel), and hydroxychloroquine. The target INR of 2 to 3 is suggested for APS patients [9]. High dose methylprednisolone pulse therapy or plasma exchange is indicated in acute life-threatening manifestations of SLE [10]. We treated our patient by the combination of plasma exchange, corticosteroids pulse therapy, and anticoagulant therapy, and the condition was improved after medication.

In conclusion, early diagnosis and adequate treatment are important to prevent multiple organ failures in SLE patients with secondary APS or severe nephritic-syndrome-related thromboembolic events. Thrombotic microangiopathy, including thrombotic thrombocytopenic purpura, hemolytic uremic syndrome, HELLP (hemolysis, elevated liver enzymes, low platelets) syndrome, and CAPS, should be considered. Higher mortality rate can be found in the patients with thrombotic microangiopathy. Aggressive treatment by anticoagulation, plasma exchange with fresh frozen plasma replacement combined with glucocorticoid pulse therapy should be performed in SLE patients with multiple venous and arterial thromboses.
